# Recruitment in an indicated prevention program for externalizing behavior - parental participation decisions

**DOI:** 10.1186/1753-2000-4-15

**Published:** 2010-05-28

**Authors:** Julia Plueck, Inez Freund-Braier, Christopher Hautmann, Gabriele Beckers, Elke Wieczorrek, Manfred Doepfner

**Affiliations:** 1 Department for Child and Adolescent Psychiatry and Psychotherapy, University of Cologne, Germany

## Abstract

**Background:**

Parents are the ones who decide whether or not to participate in parent focused prevention trials. Their decisions may be affected by internal factors (e.g., personality, attitudes, sociodemographic characteristics) or external barriers. Some of these barriers are study-related and others are intervention-related. Internal as well as external barriers are especially important at the screening stage, which aims to identify children and families at risk and for whom the indicated prevention programs are designed. Few studies have reported their screening procedure in detail or analyzed differences between participants and dropouts or predictors of dropout. Rates of participation in prevention programs are also of interest and are an important contributor to the efficacy of a prevention procedure.

**Methods:**

In this study, we analyzed the process of parent recruitment within an efficacy study of the indicated Prevention Program for Externalizing Problem behavior (PEP). We determined the retention rate at each step of the study, and examined differences between participants and dropouts/decliners. Predictors of dropout at each step were identified using logistic regression.

**Results:**

Retention rates at the different steps during the course of the trial from screening to participation in the training ranged from 63.8% (pre-test) to 81.1% (participation in more than 50% of the training sessions). Parents who dropped out of the study were characterized by having a child with lower symptom intensity by parent rating but higher ratings by teachers in most cases. Low socioeconomic status and related variables were also identified as predictors of dropout in the screening (first step) and for training intensity (last step).

**Conclusions:**

Special attention should be paid to families at increased risk for non-participation when implementing the prevention program in routine care settings.

**Trial Registration:**

ISRCTN12686222

## Background

Research literature on the prevention of children's disruptive or externalizing problem behavior provides increasing evidence for the global efficacy of multifaceted intervention packages aimed at children who are at increased risk for the development of antisocial behavior [1]. However, one specific problem in investigating such programs is the recruitment to the program itself. Different studies provide differing amounts of information about the process of recruitment. The various steps in the decision process, especially those of parents, are of particular interest because they can show that recruitment to a certain study was selective. As a result, the findings of the study would be biased according to the criteria of the CONSORT group [2], who demand transparency at every step in the reporting of randomized trials. For future trials of indicated prevention programs, as well as for the clinical implementation and dissemination of such programs, it is important to know the barriers of participation. Two main types of barrier may influence parental participation decisions:

1. Study-related barriers, which have their origin in the demands of controlled efficacy studies. Examples include the number of assessment instruments used and the time required to fill out the questionnaires, randomization, and a lack of trust in data protection procedures.

2. Intervention-related barriers, which might even influence the indication procedure (screening), as such a step is a type of intervention itself. Such barriers will be of special importance during later steps when the training itself is offered.

This study analyzes the process of recruitment within an efficacy trial of the indicated Prevention Program for Externalizing Problem behavior (PEP) [3-5]. Before describing the methods and results of our study, we review the findings of other efficacy and effectiveness studies that dealt with the same kind of problems (children's externalizing behavior) on a comparable level (indicated or secondary prevention) in young children. However, it is not easy to compare data of study flow/participation between studies because of differences such as the accuracy of data collection/report or rules of data protection for the community. Only a few studies have reported their findings of differences between participants and dropouts, or analyzed predictors of dropout.

In a study of preschool children at kindergarten, Barkley and coworkers [6] could not estimate the proportion of their sample with disruptive behavior relative to the total number of registrants. Overall, 288 of 3100 children screened via parent-rating had scores above the 93rd percentile. Also, 158 (92.9%) of the 170 parents (59.0%) who accepted the invitation to participate in the project were randomly assigned to one of the two treatment groups that included parent training. Of these 158 parents, 66.7% attended at least one session of parent training, and only 13.3% attended between 9 and 14 sessions. Comparisons between parents who did and did not attend at least one session showed that non-attendees were less well educated and rated their child's behavior as less inattentive and aggressive (using the Child Behavior Checklist, CBCL) at the initial evaluation.

Sonuga-Barke and coworkers [7] screened a total population of 3051 children at the 3-year developmental check and identified 286 children (9.4%) at risk for Attention Deficit Hyperactivity Disorder (ADHD). The parents of 105 of these children (36.7%) agreed to take part in the second step of the screening (clinical interview for ADHD) and 78 of these parents (74.3%) were included in the trial. Except for the comparison between those who declined and those who agreed to take part in the second step (slightly less severe symptoms of decliners), no findings about selectivity were reported and no information on participation was given.

In their effectiveness trial of Webster-Stratton's 10-week parenting program in a general population sample of parents, Stewart-Brown and coworkers [8] did not report details of the target sample, but mentioned a parental response rate of 69.4% for the parents of 2-8 year-old children registered with three general practices. Of the 387 parents who identified one child with worse behavior (i.e., a rating above the median of the Eyberg Child Behavior Inventory) and who were invited to enter the trial, 116 (30.0%) consented. The parents who participated did not differ from those who refused to participate in terms of their social class, but were more likely to have a child whose behavior scores were in the clinical range on the Eyberg Inventory (39.4% vs. 29.5%). The authors concluded that the approach they used seemed to reach those in need. Thirty four (56.7%) of the 60 parents in the intervention group attended at least half of the sessions, which was comparable to attendance rates in parenting programs of both high-risk or clinically-indicated samples. As the dropout rate was higher among parents of older children, the authors concluded that the optimum child age for invitation to this program was likely to be 2-3 years.

Another randomized controlled trial [9] investigated the efficacy of the Webster-Stratton 14-week group program in children (aged 2-9 years) referred for help with conduct problems (n = 158). A total of 121 primarily low-income families with parents who were able to attend group times and communicate in English met the inclusion criteria and were invited to participate in the study during a home visit by group leaders; 34 parents (28.1%) were unwilling to participate. The remaining 87 families were randomized to the intervention group (n = 44) or the wait-list group (n = 32); 11 were excluded from the analyses because they were randomized to a previously planned third arm of the study. All eligible parents who agreed to the research during the initial home visit consented to participate in the trial. Most parents of the intervention group participated in more than 5 sessions; 32 (72.7%) of 44 parents participated in 6 to 14 sessions.

Hutchings and coworkers [10] investigated the 12-week group based Webster-Stratton Incredible Years basic parenting program in a real world setting. Of 240 families with children aged 3-5 years approached by health visitors because of problem behaviors, 178 (74.2%) were contactable and interested in participating in the screening. Of these 178 families, 164 (92.1%) fulfilled the eligibility criteria and 153 (93.3%) participated in the baseline assessment interview. The authors did not discuss possible reasons for loss of families before this step of the study and conducted intention to treat analyses from the baseline assessment onwards. Mean attendance was 9.2 (SD 3.2) of 12 sessions (rate 76.7%) for the 86 participants of the intervention group (n = 104) who completed the follow-up assessments.

In the Early Risers effectiveness study, August and coworkers [11,12] investigated an indicated prevention program aimed at aggressive children and their parents or the children alone. A liberal gender-specific cutoff (t ≥ 55) was chosen. In two consecutive yearly cohorts (preschoolers and first-graders), a total sample of 2112 children was screened before obtaining informed consent. Children or parents who did not speak English sufficiently to complete the questionnaires were excluded from the study. Of the children screened, 819 (38.8%) were indicated and, of these children, 371 (45.3%) were recruited to the longitudinal study. Main reasons for loss of families were change of residence or refusal because of the possible time commitment for the intervention group. Of the families recruited, 327 were assigned to two intervention groups and 44 to a control group (normative sample). Differences between those who were retained in the study and those who officially withdrew were calculated for the course of the different interventions but not for the earlier steps of the study. No differences were found on age, gender, grade, ethnicity, IQ, female caregiver's age, SES, number of siblings living with the child and, most importantly, severity of initial aggressive behavior. However, there were more retained children who came from single parent households. Dropouts from the child-intervention-only group had significantly higher aggression scores than those from the control group.

Treatment barriers have also been analyzed in universal prevention studies. Heinrichs and coworkers [13] analyzed barriers to research and program participation in a universal prevention program (Triple P) for child behavior problems in Germany. They reported a target sample of 915 eligible participants; 282 families (30.8%) were enrolled in the project. Analyses of the social structure within the sample, determined by an objective kindergarten social structure index (OKS), showed that preschool areas with few social structure problems (high OKS) were overrepresented compared to areas with moderate or low OKS. Because each preschool teacher team was asked to rate each family in their group on a number of sociodemographic variables, it was possible to analyze reasons for attrition. Logistic regression showed that parents from single-parent homes were 1.56 times more likely to participate after controlling for occupation, social status, number of family members and parental age. Parents with low or medium SES were less likely to participate after controlling for other variables. Forty percent of the non-participating families answered questions about their reasons for non-participation and mainly reported assessment-related barriers, such as intrusion of their privacy, as their primary concern (pretest at home visit). Of the186 families randomized to the intervention group, 144 (77.4%) attended at least one session; most families (89.0%) participated in three or four sessions. Logistic regression of predictors for non-participation (controlling for other variables) found a higher risk for single-parent families and families with low SES, whereas parents who described more externalizing problems were more likely to participate in the training.

In this paper, we focus on parental decisions on participation at each step of the efficacy study of the indicated Prevention program for Externalizing Problem behavior from recruitment to the intervention phase. At each step of the study, we determined the retention rate, examined differences between participants and dropouts/decliners, and identified predictors of non-participation.

## Methods

### Study course

**Figure 1:**
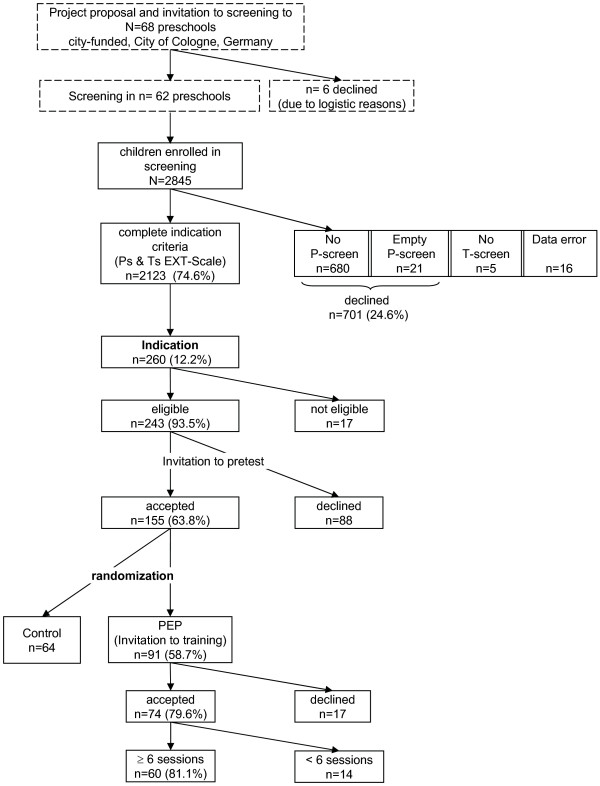
**Course of recruitment in the effectiveness study evaluating the PEP**.

Figure 1 gives an overview of the various steps of the study, which has been described in more detail elsewhere [4,5]. In summary, there was a screening, identification of those indicated and eligible for treatment, a pre-test assessment, and randomization to training.

Public preschools in a German city of about 1,000,000 inhabitants served as the primary recruitment sites and were selected in cooperation with the Department of Youth Welfare of the city. The randomized control group trial for an indicated prevention program required a screening procedure to select the target group. At this step, participation was anonymous and parents could decline to take part at this and any subsequent steps of the study if they were not interested in receiving feedback on the findings of the screening or if they did not want to participate any further. All screening participants who gave consent received a letter with a summarized feedback of the screening and those indicated were informed that project staff would telephone them within the next two weeks to tell them about the pre-test assessment, ask for consent, and to fix a date for the assessment. The pre-test included a booklet of questionnaires for both the mother and father, and a home visit lasting up to 3 hours (with intelligence testing of the child, an interview with at least one parent, and a videotaped standardized interaction task with one parent and the child). As compensation for their time and effort, parents were offered €30 for the home visit and an additional €20 for completing the parents' questionnaires.

### Measures

Information that could be used to identify reasons for refusal to participate, especially during the early steps of the process, could be taken from the screening instrument (PEP-Screen, see additional file 1 and 2), which has been described in detail elsewhere [14]. Similar to the study of the Conduct Problems Prevention Research Group [15,16], our screening used 13 items taken from the German version of the Child Behavior Checklist 4 to 18 [17,18], which assesses behavioral and emotional problems using a 3-point scale (0 = "not true", 1 = "sometimes or somewhat true", 2 = "exactly/often true"). An externalizing behavior score was empirically confirmed by factor analyses and showed satisfactory internal consistency (r_it _= 0.74-0.89). It was calculated from the sum of scores for the following 7 items: item 1 (argues a lot); item 5 (can't concentrate); item 6 (can't sit still or is hyperactive); item 8 (destroys things belonging to others); item 10 (impulsive or acts without thinking); item 12 (physically attacks others); and item 13 (temper tantrums). For co-morbid internalizing problems, items 4 (clings to or is too dependent to adults), 7 (too fearful or anxious), 9 (unhappy or sad), and 11 (pain without good somatic reason) were included. Item 2 (getting teased a lot) and item 3 (demands too much attention) remained in the questionnaire, but only counted in the total score. The sum of parents' and teachers' ratings of the externalizing score was used as the indication criterion with a cut-off at the 88^th ^percentile of the screening-sample (which was the closest raw-score to the 85^th ^percentile which we intended to use for cut-off). In addition, the parents' and teachers' version of the PEP-screen had two global questions for an overall rating of the child's problems: (1) "How much do you feel bothered/burdened by the child's behavior?" rated as No, Yes a bit, Yes medium, or Yes a lot, and dichotomized as yes/no for the logistic regression analyses; and (2) "Do you think you or the child need(s) professional help because of the burden?" rated as Yes or No.

When parents did not participate in the screening, some demographic information was available from the teachers' screening (age and gender of the child, parents' language (German/others)). Moreover, in a multiple-choice question, teachers were asked to assess parents' and their own view on the reasons for the parents' decision not to participate (language problems, concerns about data protection, additional free answers). Based on information obtained from the parents' screening questionnaire, SES was estimated as mean of education and profession of both parents, and classified as high, medium or low. Data from the pre-test assessment was another source of information for the analysis of parental interest and participation intensity in the training.

Ethical concerns about using data from families who did not give consent were taken into account by using only the teacher's information about the child's behavior in preschool. Information about the family was only taken from the parents themselves when consent was given.

The independent variables describe the behavior of the child or sociodemographic characteristics of the child or family and, therefore, are representative of the internal factors for parents declining. The external barriers (study- or program-related) in these analyses are represented by the different steps of the recruitment procedure (up to pre-test, beginning of the prevention program).

### Intervention

The intervention consisted of two components: a parent training and a teacher training of 10 sessions each (one session of 90 to 120 min per week) conducted by a psychologist with special training in this intervention. Parents were told that the trainer worked with groups of up to 6 participants, with separate sessions for parents and teachers usually during preschool time, but that a different time and place could be arranged depending on individual needs as far as possible. Moreover, parents were told that homework assignments (practicing strategies individually planned during the sessions) were part of the training, which lasted up to three months and was followed by a post-test.

### Statistical analysis

To analyze the parental decisions, all ranked variables were dichotomized. At every step, the differences between participants and dropouts/decliners for available variables were calculated using t-tests for continuous variables, and χ^2 ^tests for categorical variables. All variables available were included in a stepwise logistic regression analysis to determine the set of variables associated with participation versus non-participation at each step of the study.

Because significance testing in stepwise logistic regression is of questionable reliability, the analyses were repeated using the "enter" method. That is, all independent variables identified in the stepwise analysis were entered into the model at the same time and the Odds Ratio (OR) of each variable in the model was estimated (including the 95%confidence interval, CI) and the reduction of the 2-Log-Likelihood was tested for significance (χ^2^-Likelihood-Ratio-Test) [19]. The goodness of fit of the entire model was tested using the Hosmer-Lemeshow chi-square test. Well-fitting models show non-significance on this test, indicating model prediction is not significantly different from observed values. In addition, statistical (non-)significance of the model does not mean that it necessarily explains much of the variance in the dependent variable only that however much it does explain is more than random. Therefore, odds ratio of the variables in the model will be reported as a descriptive measure. Moreover, if the sample gets smaller, as occurs during the course of the project, the test may overestimate the model fit [20].

## Results

The course of recruitment and retention during the efficacy trial, including screening, indication, pre-test, randomization, and training is shown in Figure 1. Sixty eight city-funded preschools in the city of Cologne, Germany, were selected by project staff with the aim of having nearly equal representation of districts with different levels of social burden, an indicator supplied by the Department of Youth Welfare of the city integrating different aspects of social burden and need for public youth welfare. Six preschools were excluded for logistic reasons, such as planned closing, rebuilding or planned changes of staff within the next months. For the remaining 62 preschools, the screening procedure focused on children aged 3 to 6 years who were expected to stay in preschool for at least one year and, therefore, would be applicable for the subsequent steps of the project at least up to the post-test immediately after the training.

### Screening

In the screening procedure, parents could choose between three different levels of participation: (a) to assess their child via the screening questionnaire and give consent for the teacher to forward their name and address to project staff for later contact; (b) to complete the assessment but to remain anonymous; or (c) not to assess their child at all. Teachers collected parents' questionnaires and assessed the children themselves using the teachers' version of the questionnaire.

A sample of n = 2845 children was assessed by at least one adult (parent or teacher). Data protection issues prevented us from checking the accuracy of the size of our target sample, but we consider it to be good because the only reason not to include a child at this step was a long lasting absence from preschool despite being formally enrolled. Half of the sample (50.2%) were boys, the mean age was 4.08 years (SD = 0.86), and different areas of social burden were represented equally. Parents of 2123 (74.6%) children actively participated in the screening procedure. For parents who declined to participate (n = 701), the teachers' information identified that the main language of the declining parents was German for 31.2% and another language for 44.1%; no information concerning language was available for the remaining 24.7%. Of the declining parents, 6.3% mentioned language problems as a reason for their refusal, but teachers suspected this reason in 19.0% of cases. Concerns about data protection were reported by 4.9% of parents and 6.3% of teachers.

At this first step of the analysis of parents' decisions, children whose parents agreed to participate (n = 2123) and those whose parents declined participation in the screening (n = 701) - either actively (empty questionnaire) or passively (no feedback at all) - were compared using the available information from the teacher's screening: gender and age of the child, index of social burden of the district the preschool belonged to, teacher's burden by the child's problems, teacher's need for help because of these problems, and aggregated scales for internalizing and externalizing behavior, as well as the total score from the screening questionnaire.

As Table 1 shows, parents who declined to participate in screening had older children and came from districts with a higher social burden compared with those who participated in the screening. The two groups did not differ significantly on externalizing, internalizing and total problem scores on the teacher screening checklist, and girls and boys were distributed equally. However, for the group that declined screening, teachers reported more need for help.

**Table 1:** Comparison between participants and non-participants at the early steps of the study

Potential predictors	Screening						Eligible						Pre-Test					
	Participant N= 2123		Decliner n = 701		statistics		Participant N= 243		Decliner n = 17		Statistics		Participant N= 155		Decliner n = 88		statistics	
	**mean**	**sd**	**mean**	**sd**	***p *_(t-test)_**	**OR**^**a**^	**mean**	**sd**	**mean**	**sd**	***p *_(t-test)_**	**OR**^**a**^	**mean**	**sd**	**mean**	**sd**	***p *_(t-test)_**	**OR**^**a**^

Age (Child)	4.06	0.86	4.15	0.84	0.015*		4.17	0.85	4.24	0.97	0.757		4.18	0.87	4.15	0.81	0.772	
SES^b^	-	-	-	-	-		1.25	0.74	1.33	1.00	0.690		1.31	0.74	1.15	0.74	0.121	
Ts-ext	3.12	3.58	3.40	3.69	0.079		9.87	2.66	10.76	2.54	0.181		9.57	2.76	10.40	2.40	0.020*	
Ts-int	1.45	1.60	1.45	1.65	0.950		1.62	1.61	1.88	2.06	0.527		1.49	1.53	1.85	1.73	0.093	
Ts-total	5.46	4.59	5.75	4.81	0.157		13.12	4.00	14.24	4.21	0.270		12.66	3.89	13.93	4.09	0.017*	
Ps-ext	-	-	-	-	-		7.42	2.63	5.94	2.44	0.025*		7.76	2.49	6.83	2.78	0.008**	
Ps-int	-	-	-	-	-		1.90	1.67	1.35	1.06	0.187		1.97	1.75	1.76	1.51	0.341	
Ps-total	-	-	-	-	-		11.02	4.00	9.24	3.63	0.076		11.49	3.90	10.21	4.06	0.018*	

	**%**		**%**		***p *(χ**^**2**^**)**	**OR**	**%**		**%**		***p *(χ**^**2**^**)**		**%**		**%**		***p *(χ**^**2**^**)**	**OR**

Gender (male)	49.9		51.1		0.623		74.1		70.6		0.752		72.9		76.1		0.580	
SBD^c^	**37.6**		**50.9**		0.000**	**1.69**	44.4		35.3		0.462		40.0		52.3		0.064	
Ts burden by child	15.0		17.3		0.150		64.6		76.5		0.320		63.2		67.0		0.550	
Ts need for help	**19.7**		**25.0**		0.003**	**1.34**	70.4		70.6		0.988		71.2		69.0		0.710	
Ps burden by child	-		-		-		38.3		17.6		0.088		**47.1**		**23.0**		0.000**	**.34**
Ps need for help	-		-		-		**42.6**		**11.8**		0.019*	**.18**	49.3		30.6		0.005**	

^a^OR = Odds Ratio (amount or increase in odds for decline with increasing values of the predictor); only if *p *≤ 0.05 or for identified predictors in the logistic regression equation

^b^SES = Socio Economic Status (calculated as mean of both parent's education and profession).

^c^SBD = Social Burden of District (composite indicator calculated by the department of youth and family welfare of the City of Cologne) (very low, low, neither/nor, burden, strong burden); range in each sample '-2' to '2' dichotomized in: no or average SBD vs. moderate to high SBD

Ts = Teachers view in screening; Ps = Parents view in screening; int- = Internalizing-score; ext = Externalizing-score; tot = Total-score

* *p *≤ 0.05; **p ≤ 0.01; significant (2-tailed) differences between participants and decliners at early steps of decision concerning project participation and indication for prevention,

- Unattainable

Bold characters: variables included in the logistic regression model

In the stepwise logistic regression analysis, SBD (OR = 1.69; CI = 1.42-2.01) and teacher's need for help (OR = 1.34; CI = 1.09-1.64) were included in the model. The 2-log-likelihood indicated a good model fit (3129.67-3085.75; *p ≤ *.05) and the non-significant Hosmer-Lemeshow test (χ^2 ^= 1.83; df = 2, *p*=.400) indicated no meaningful difference between observed and model-predicted values; therefore, improvement of the classification by including the identified variables in the model could be verified.

### Indication - eligibility

Of the children screened, 260 (12.2%) were defined as being at risk for developing more severe problems and indicated for the intervention (see Figure 1). Mean age of these children was 4.17 years (SD = 0.85), 73.8% were boys, and the different areas of social burden were represented equally. Of the indicated families, 243 (93.5%) agreed to their address being forwarded and were defined as eligible. Table 1 presents the variables that were statistically significantly different between the participant and decliner groups at this step of the decision process, and includes both teachers' and parents' information from screening. Parents who declined reported less externalizing problems and less need for help compared with participants. In the stepwise logistic regression, only the parental need for help (OR= 0.18; CI= 0.04-0.81) was included in the model. The 2-log-likelihood significantly decreased (from 124.36 to 117.17, *p ≤ *.05) but the Hosmer-Lemeshow chi-square test could not be performed for the total model because of the small number of decliners.

### Pre-test

One hundred and fifty five families (63.8% of those eligible) agreed to participate in the pre-test step of the study; 22.1% were single-parent families and mean age of the mothers was 33.26 years (SD = 6.03). Because this information was only available for participants, comparison with those who declined was not possible. Variables that were significantly different between the two groups at pre-test are summarized in Table 1. Declining parents (n = 88) rated their child's behavior (at screening) as showing less externalizing problems compared with participating parents, and (therefore) felt less burden and less need for help. In contrast, teachers' ratings of children's behavior showed higher ratings in externalizing and total problems in the declining group, but no significant difference in their felt burden or need for help.

In the stepwise logistic regression, only parents' burden by child (OR= 0.34; CI= 0.19-0.61) was included in the model. The 2-log-likelihood significantly decreased (from 314.33 to 300.19; *p *≤ .05) by including the variable, and the Hosmer-Lemeshow chi-square test could not be performed for the total model.

### Readiness for training

Participants of the pre-test were randomly assigned to the training and control groups with oversampling for the intervention group using a 3:2-ratio to maintain a large sample of combined parent and teacher training for the efficacy analyses. Thus, 91 (58.7%) families and teachers were defined as the intervention group and received an offer to participate in the training. Children's mean age was 4.20 years (SD = 0.85) and 74.7% were boys. The different areas of social burden were distributed nearly equally in the intervention group, mean SES was 0.72 (SD = 0.72), 25.3% lived in single-parent families, and mean age of the mother was 32.80 years (SD = 6.23). From this step on, teachers could participate in training independently from parents' decision. Parents of 74 children accepted participation in the training and attended at least one of the 10 sessions.

The first section of Table 2 lists the variables available for comparison between parents willing to participate and those who declined to participate in the training. Children of declining parents (n = 17) were rated by their parents as showing less externalizing behavior problems in the pre-test (CBCL) and teachers felt less burden by those children in screening. In the stepwise logistic regression, children's internalizing behavior (screening) and externalizing behavior (pretest) as well as gender were included in the model. The odds ratios calculated within the confirming logistic regression (using enter method) show that parents who described more externalizing behavior were less likely to decline participation in training (OR= .88; CI= .80-.96), while parents describing higher rates of internalizing behavior in an earlier step (screening) were more likely to refuse training (OR= 2.00; CI= 1.30-3.08). Parents of boys were also more likely to decline participation at this step (OR= .08; CI=.01-.79). The indicators for the quality of the model were good: the classification of cases slightly improved (from 80.7% to 84.1%), the 2-log-likelihood significantly decreased (from 86.38 to 63.18 *p *≤ .05), and the model fit was good as indicated by a non-significant difference of observed and predicted values on the Hosmer-Lemeshow test (χ^2 ^= 3.09; df = 8; *p*=.928).

**Table 2:** Comparison between participants and non-participants at the later steps of the study

Potential predictors	Readiness for training						Training intensity					
	Yes N= 74		No N = 17		statistics		Frequent N = 60		Rare N = 14		statistics	
	**mean**	**sd**	**mean**	**sd**	***p *_(t-test)_**	**OR**^a^	**mean**	**Sd**	**mean**	**sd**	***p *_(t-test)_**	**OR**

Age (Child)	4.19	0.87	4.24	0.75	0.841		4.12	0.87	4.50	0.85	0.139	
Age (mother)	33.41	6.23	30.29	5.75	0.063		33.93	4.69	31.14	10.55	0.349	
SES^b^	1.35	0.75	1.34	0.75	0.956		**1.48**	**0.74**	**0.79**	**0.50**	0.001**	**.25**
Ts-ext	9.88	2.63	9.35	2.67	0.460		9.77	2.53	10.36	3.05	0.453	
Ts-int	1.59	1.56	1.18	1.42	0.315		1.40	1.45	2.43	1.79	0.025*	
Ts-tot	13.03	3.45	12.12	4.20	0.350		12.68	3.30	14.62	3.80	0.067	
Ps-ext	7.61	2.47	7.59	2.45	0.976		7.47	2.56	8.21	2.01	0.311	
Ps-int	**1.82**	**1.56**	**3.00**	**2.47**	**0.076**	**2.00**	1.82	1.59	1.86	1.51	0.931	
Ps-tot	11.13	3.86	12.50	4.00	0.206		10.95	4.02	11.86	3.13	0.434	
C-TRF-ext	29.71	12.85	26.81	12.72	0.416		12.59	7.90	13.69	8.01	0.652	
C-TRF-int	12.79	7.88	9.94	4.58	0.167		29.14	12.94	32.31	12.55	0.424	
C-TRF-tot	54.21	24.45	46.00	17.93	0.209		53.05	24.85	59.46	22.69	0.396	
CBCL-ext	**20.61**	**8.67**	**15.94**	**7.36**	0.044*	**.88**	12.32	7.51	13.79	8.99	0.530	
CBCL-int	12.61	7.78	12.88	9.56	0.900		20.47	8.82	21.14	8.34	0.798	
CBCL-tot	44.41	20.76	37.94	22.38	0.259		43.98	21.22	46.14	19.44	0.730	

	**%**		**%**		***p *(χ**^**2**^**)**	**OR**	**%**		**%**		***p *(χ**^**2**^**)**	**OR**

gender (male)	**70.3**		**94.1**		**0.061**	**.08**	73.3		57.1		0.233	
SBD^c^	31.1		47.1		0.210		28.3		42.9		0.290	
Single-parent	25.7		23.5		0.854		25.0		28.6		0.746	
Ts-burden by child	73.0		41.2		0.012*		**70.0**		**85.7**		**0.326**	**(3.04)**
Ts-need for help	82.2		76.5		0.731		78.0		100.0		0.061	
Ps-burden by child	47.9		52.9		0.711		49.2		42.9		0.672	
Ps-need for help	51.4		35.3		0.232		51.7		50.0		0.908	

^a^OR = Odds Ratio (amount or increase in odds for decline with increasing values of the predictor); only if *p *≤ 0.05 or for identified predictors in the logistic regression equation

^b^SES = Socio Economic Status (calculated as mean of both parent's education and profession).

^c^SBD = Social Burden of District (composite indicator calculated by the department of youth and family welfare of the City of Cologne) (very low, low, neither/nor, burden, strong burden); range in each sample '-2' to '2' dichotomized in: no or average SBD vs. moderate to high SBD

Ts = Teachers view in screening; Ps = Parents view in screening; C-TRF = Teachers view, pretest; CBCL = parents view, pretest; int- = Internalizing-score; ext = Externalizing-score; tot = Total-score

* *p *≤ 0.05; **p ≤ 0.01; significant (2-tailed) differences between participants and decliners at steps of decision concerning training

Bold characters: variables included in the logistic regression model

**Figure 2:**
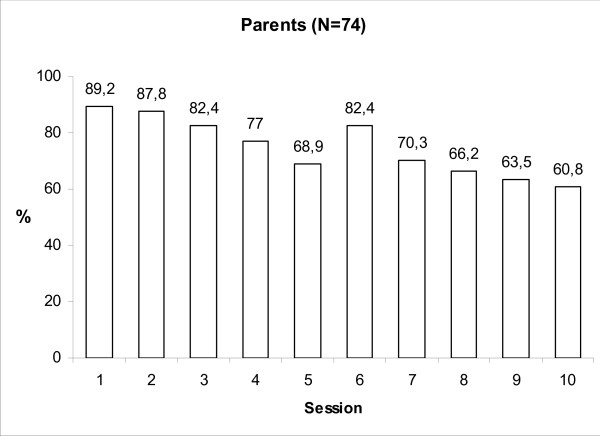
**Parents participation rates per session**.

**Figure 3:**
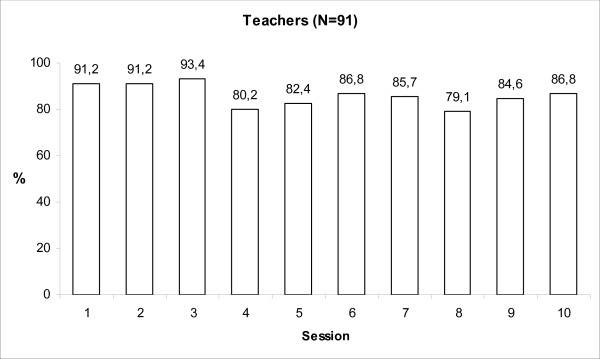
**Teachers participation rates per session**.

The parents' mean participation rate per training session was 74.9% and Figure 2 shows a slight decrease during the course of the training from 89.2% (session 1) to 60.8% (session 10). The mean number of sessions attended by parents was 7.5 (SD = 2.7). The corresponding figures for the 91 teachers participating in at least one session were a mean participation rate of 86.1%, ranging from 93.4% for session 3 to 79.1% for session 8 (Figure 3). A mean number of 8.8 (SD = 1.8) sessions was attended by teachers.

### Training intensity

Table 2 also lists the variables that were significantly different between those parents who took part in at least 6 of the 10 group training sessions (n = 60) and those who participated in fewer sessions (n = 14). The families who participated less showed a significantly lower SES and the children were rated significantly higher on the screening scale for internalizing behavior by their teachers. In the stepwise logistic regression, SES and teacher's burden by the child were included in the model. Only SES showed an odds ratio significantly different from 1. Therefore, the confirming logistic regression ("enter" method) was carried out with this variable alone (OR= .25 CI= .10-.64). The significantly decreasing 2-log-likelihood (from 71.36 to 61.05; *p *≤ .05) as well as the non-significant Hosmer-Lemeshow test (χ^2 ^= 9.69; df = 8; *p*=.288) indicating no meaningful difference between observed and model-predicted values referred to a good model fit of the total model.

## Discussion

Analysis of participation rates at different steps of the decision process may be useful to document the course of recruitment and get hints on the generalizability of the findings of the efficacy study. The main objective of this study was to get information on barriers of participation as far as the data available allowed in a randomized clinical trial. This information may be useful for optimizing recruitment procedures for an indicated prevention program. We expected study-related barriers (e.g., investment of time for assessment) to be important in the decision of whether or not to participate in the screening process and in the pre-test assessment. In addition, intervention-related barriers (e.g., confrontation with family problems, time for participating in the training sessions) might be of special importance for the steps following the offer of training. Certainly, we can only compare the rates of decliners within these two sections of the trial. The highest attrition rates were observed for the screening and the subsequent pre-test assessment: one-quarter of all parents invited declined the screening and more than one-third declined the invitation to the pre-test. Since screening is a necessary step in an indicated prevention program, the extended pre-test in this trial can be interpreted as a study-related barrier. In the last step, 20% of parents decided not to take part in the training offered, whereas 80% of those who initiated the training attended more that 50% of the l0 sessions provided.

In this trial of the efficacy of an indicated prevention program for children with externalizing behavior problems, nearly 75% of the community sample participated in the initial screening. Findings from epidemiological studies and a few studies similar to the present one (69.4% [8]; 74.2% [10]) suggest this rate can be considered satisfactory. However, the analyses of predictors for declining participation in the screening procedure showed that living in districts with a higher social burden and a higher need for help described by the teacher increases the odds of declining. This indicates that a substantial group of parents with children at risk for externalizing behavior problems was missing at the screening step.

Only a small proportion of parents whose children were indicated were not eligible because they had not given their address (16.5%), but these parents (who were not interested in feedback) described less need for help than parents who participated.

The highest attrition occurred at the pre-test, where more than one-third of the sample declined participation. Other studies starting with screening of a community sample have reported similar or higher figures, ranging from 28.1% [9] to 45.3% [12]. The only factor predicting attrition at the pre-test step was a reduced burden by their child as perceived by the parents. In agreement with Stewart-Brown and coworkers [8], we can conclude that our approach seems to reach those (more) in need based on parents' perception. However, from the teachers' perspective, there were trends in the opposite direction (higher scores in aggressive behavior and total behavior problems in those who declined), which may be partly due to a methodological artifact because patients with lower scores in parent's rating must have higher scores in teacher's rating in order to fulfill the indication criterion of combined parent and teacher ratings. The differences in parent and teacher ratings may reflect real differences in behavior problems in the different setting or they may reflect a rater bias.

In the first case the higher attrition rate of the parents can be interpreted as a consequence of a reduced need for help. In the second case parents are in need for help but they refuse it since they do not consider their child's behavior as problematic due to a rater bias. In these cases the first step of a successful prevention program would be to increase the problem perception of the parents for example by discussing the different perspectives of the parents and the teachers.

In agreement with Gardner and coworkers [9], we found lower rates of decliners after the pre-test. Therefore, we can support their conclusion that "when you reach to get behind the doorstep it is much more probable that the families take part in the next steps". Nearly 80% of the invited parents participated in at least one session of the training (and most of them more), which is better than the 66.7% participation rate reported by Barkley and coworkers [6]. But is has to be taken in account that the indication criterion in this study tried to identify a more "clinical" externalizing sample, not only children at risk. Studies comparable to ours did not report basic participation rates for training. Parents were more likely to decline participation in training if they identified less externalizing behavior problems in their child or described more internalizing problems at the screening step. The effect of gender on likelihood for participation in training was small and probably not clinically relevant.

The results on readiness for training may be interpreted as those who especially need help from an indicated prevention program for externalizing problem behavior are likely to be included. Some findings of other studies show that this is not self-evident [11,12]. Our results on child symptom intensity correspond to those of Barkley and coworkers [6] but, in contrast to these authors, we did not find that parents' education or other SES-related variables were predictive of parent compliance in starting treatment (i.e., readiness for training). At this step one can assume that the main study-related barriers had already been overcome, whereas parental decisions about participating may be influenced by the training itself and related reasons (program-related barriers).

A high proportion of parents regularly participated in the training (took part in ≥6 sessions), which is comparable to other well implemented programs (e.g., Webster-Stratton's "Incredible Years" [8,10]). The teachers' participation rate in the training was consistently higher than that of parents, but teachers could participate during their work time. Moreover, the program dealt with their professional and not their private/personal circumstances. The finding that families of lower SES had more problems in regular participation is consistent with that of Heinrichs and co-workers [13] in their investigation of universal prevention. It also corresponds to our finding that SBD (which may be an indicator of SES) correlates with screening participation in the first step of our analysis. That is, parents with a lower SES have a higher risk of declining screening and of less frequent participation in the treatment process compared with parents with a higher SES. Therefore, trainers should be aware that lower SES parents may need extra support to continue with the training. Individual reasons for missing a single session were not investigated systematically but may be associated with problems in practical organization (e.g., time, health, transport), attitudes towards the training (rank of importance), or experiences with the training (i.e., boring, not helpful, difficult). As Heinrichs showed in a trial with families from social disadvantaged areas focusing on different ways of recruitment, payment for participation was helpful in increasing the participation rates in a universal prevention program [21].

Satisfactory rates of participation in training showed that the program itself is well accepted, but the association with SES is alarming and sends an important message to trainers to pay special attention towards keeping low SES parents in the program.

Moreover, teachers of children whose parents showed up to the sessions with less frequency more often reported need for help. This is related to the lower participation rates of parents of children with lower problems as rated by parents but higher problem scores as rated by teachers at pre-test.

### Limitations

The results of these analyses are influenced by the criterion we used for indication. The combination of parent and teacher ratings was used to identify children with the highest risk. An alternative definition of this criterion (i.e., high scores in both settings) may have led to other results.

In contrast to Heinrichs and coworkers [13], our analyses focused on variables gathered in the "natural" process of data collection. For project economic reasons, we only used a reduced "special" dropout questionnaire and did not compel the teachers to answer questions about parents not participating in screening. For the same reason, we did not systematically ask parents declining at each of the subsequent steps, especially pre-test and training participation, for their specific reasons for declining.

At least one variable was included in the logistic regression model at each step. For some models it was not possible to calculate the goodness-of-fit tests. However, statistical (non-)significance alone might not be sufficient for defining important predictors because the sample size was quite large at least at the first steps. However, the ORs were low, indicating that other factors might be more important in explaining parental participation decisions.

## Conclusions

The attrition rate over the course of the study was substantial but comparable to other studies. Study-related and program-related barriers for participation were identified. For the variables available, attrition rates could only be explained to a small extent. Variables known to increase the risk for development of a disorder (i.e., SES, SBD) also increased the risk for non-participation. Furthermore a special task might be to motivate parents for participation who themselves suffer less, although their children showed externalizing problems as reported by their teacher. Thus, interventions to raise participation rates in prevention programs are important, with particular attention being paid to families with a lower SES. Although in our study this variable was only identified on the level of training intensity, SES may be important at different recruitment steps of indicated or selective prevention programmes. Lower SES may increase several barriers for participation and therefore several aspects of "practical organisation" should be addressed systematically in future research on dissemination especially of parent focussed programmes. As Kumpfer [22] already pointed out engagement, recruitment and retention strategies should also be carried out removing such attendance barriers (e.g. meals, child care, transportation and incentives for homework completion).

## List of abbreviations

ADHD: Attention Deficit Hyperactivity Disorder; PEP: Prevention program for Externalizing Problem behavior; OR: Odds Ratio (amount or increase in odds for decline with increasing values of the predictor); only if *p *≤ 0.05 or for identified predictors in the logistic regression equation; SES: Socio Economic Status (calculated as mean of both parent's education and profession); SBD: Social Burden of District (composite indicator calculated by the department of youth and family welfare of the City of Cologne) (very low, low, neither/nor, burden, strong burden); range in each sample '-2' to '2' dichotomized in: no or average SBD vs. moderate to high SBD; C-TRF: Caregiver-Teacher Report Form 1 1/2-5, teacher's view, pretest; CBCL: Child Behavior Checklist 1 1/2-5; parent's view, pretest; Ts: Teacher's view in screening; Ps: Parent's view in screening; int: Internalizing-score; ext: Externalizing-score; tot: Total-score.

## Competing interests

Dr. Doepfner is Head of the School for Child Behavior Therapy

## Authors' contributions

All authors were part of the PEP-Team who carried out the efficacy study of the Prevention Program of Externalizing Problem behavior (PEP). All authors except MD participated in data collection and carried out the parent group trainings. JP and MD worked together in analyzing the results, interpreting the findings and developing the manuscript, including writing and editing. All authors read and approved the manuscript prior to submission.

## Supplementary Material

Additional file 1 **PEP-Screen-EL**. Questionnaire used for screening of children at risk for externalizing behavior, parents' perspective. Click here for file

Additional file 2 **PEP-Screen-ER**. Questionnaire used for screening of children at risk for externalizing behavior, teachers' perspective. Click here for file
