# Supplementary material for: Recruitment in an indicated prevention program for externalizing behavior - parental participation decisions
Source: Child Adolesc Psychiatry Ment Health. 2010 May 28;4:15. doi: 10.1186/1753-2000-4-15 (PMC2897776; doi:10.1186/1753-2000-4-15)
Supplement: Additional file 1 — PEP-Screen-EL. Questionnaire used for screening of children at risk for externalizing behavior, parents' perspective. [file 1753-2000-4-15-S1.PDF]

ELTERNBEFRAGUNG

Liebe Eltern,

Ihr Kindergarten nimmt an einer **Studie zur Früherkennung und Vorbeugung von Verhaltensproblemen** teil, die von der Universität zu Köln in Kooperation mit dem Jugendamt Köln durchgeführt wird. Um festzustellen, wieviel Unterstützung dieser Art nötig ist, brauchen wir **Ihre Mithilfe!** Mit dem vorliegenden **kurzen Fragebogen** möchten wir an einer möglichst großen Gruppe von Kindern im Kindergartenalter ermitteln, wie oft bestimmte Probleme nach der Einschätzung der Eltern und ErzieherInnen vorkommen. Die Befragung hängt also entscheidend auch von Ihrer Beteiligung ab, denn jeder einzelne Bogen ist wichtig! Also:

Machen Sie mit!
Nehmen Sie sich bitte ein paar Minuten Zeit und füllen Sie den Fragebogen aus.
Geben Sie ihn dann möglichst bald im verschlossenen Umschlag im Kindergarten wieder ab.

Die Befragung ist anonym, d.h. nur die ErzieherInnen kennen die Identifikationsnummer des einzelnen Kindes, haben aber keine Einsicht in Ihren Fragebogen. Angaben zu den Eltern selbst dienen lediglich der Beschreibung unserer Stichprobe. Da Sie sich jedoch die Mühe machen, den Fragebogen auszufüllen, möchten Sie sicher auch wissen, was die Befragung ergeben hat.

Grundsätzlich sollen daher alle Eltern eine Rückmeldung erhalten.

Zu diesem Zweck ist es nötig, daß die ErzieherInnen Ihre Anschrift an uns weitergeben können. Sollten Sie an einer solchen Rückmeldung jedoch nicht interessiert sein, können sie dies am Ende des Fragebogens (s. Rückseite) angeben.

Fragebogen für Eltern von Kindergartenkindern

IDENTIFIKATIONSNUMMER (des Kindes):
(Wird durch ErzieherIn vergeben)
|_|_|_|_|_|

GESCHLECHT: ☐ Junge ☐ Mädchen

ALTER DES KINDES:

GEBURTSTAG DES KINDES: |_|_|_|_|_|_|
Tag Monat Jahr

HEUTIGES DATUM: |_|_|_|_|_|_|
Tag Monat Jahr

DIESER FRAGEBOGEN WURDE AUSGEFÜLLT VON :

☐ Mutter ☐ Vater
☐ Andere (genaue Bezeichnung):_______________

SCHULAUSBILDUNG DER ELTERN :

Mutter	Vater	
☐	☐	Kein Abschluß
☐	☐	Hauptschulabschluß
☐	☐	Realschulabschluß
☐	☐	Fach-/Hochschulreife/Abitur

BERUFSAUSBILDUNG DER ELTERN :

Mutter	Vater	
☐	☐	Un-/ Angelernt
☐	☐	Abgeschlossene Berufsausbildung
☐	☐	Abgeschlossenes Studium

! Bitte wenden !

Bitte füllen sie diesen Fragebogen so aus, daß er Ihre Ansichten wiedergibt, auch wenn andere Menschen diese nicht teilen. Weitere Bemerkungen oder Kommentare können Sie gerne auf ein extra Blatt schreiben.

Es folgt eine kurze Liste von Eigenschaften, mit denen man Verhalten von Kindern beschreiben kann. Beantworten Sie bitte für jede Eigenschaft, ob sie jetzt oder innerhalb der letzten 2 Monate bei dem Kind zu beobachten war. Wenn diese Eigenschaft sehr deutlich oder sehr oft zu beobachten war, kreuzen Sie die Ziffer 2 an, wenn die Eigenschaft etwas oder manchmal auftrat, die Ziffer 1, wenn sie für das Kind nicht zutrifft, die Ziffer 0. Beantworten Sie bitte alle Fragen so gut Sie können.

0= nicht zutreffend (soweit bekannt)	1= etwas oder manchmal zutreffend	2= genau oder häufig zutreffend

1. Streitet oder widerspricht viel	**0**	**1**	**2**
2. Wird viel gehänselt	**0**	**1**	**2**
3. Verlangt viel Beachtung	**0**	**1**	**2**
4. Klammert sich an Erwachsene oder ist zu abhängig	**0**	**1**	**2**
5. Kann sich nicht konzentrieren, kann nicht lange aufpassen	**0**	**1**	**2**
6. Kann nicht stillsitzen, ist unruhig oder überaktiv	**0**	**1**	**2**
7. Ist zu furchtsam oder ängstlich	**0**	**1**	**2**
8. Macht Sachen kaputt, die anderen gehören	**0**	**1**	**2**
9. Ist unglücklich, traurig oder niedergeschlagen	**0**	**1**	**2**
10. Ist impulsiv oder handelt ohne zu überlegen	**0**	**1**	**2**
11. Sagt häufig, daß ihm etwas weh tut (ohne bekannte körperliche Ursache)	**0**	**1**	**2**
12. Greift andere körperlich an	**0**	**1**	**2**
13. Hat Wutausbrüche oder hitziges Temperament	**0**	**1**	**2**

Insgesamt gesehen,
als wie belastend erleben Sie die oben beschriebenen Verhaltensweisen bei Ihrem Kind?
(Zutreffendes bitte ankreuzen)

☐ gar nicht ☐ ein wenig ☐ ziemlich ☐ sehr

Glauben Sie, daß Ihr Kind wegen dieser Probleme eine Beratung oder Behandlung braucht, oder daß Sie sich deswegen beraten lassen sollten?

☐ Nein ☐ Ja

BITTE PRÜFEN SIE, OB SIE ALLE FRAGEN VOLLSTÄNDIG BEANTWORTET HABEN! **VIELEN DANK !**

☐ Nein, bitte keine Rückmeldung über die Ergebnisse dieser Befragung.
