# Supplementary material for: Recruitment in an indicated prevention program for externalizing behavior - parental participation decisions
Source: Child Adolesc Psychiatry Ment Health. 2010 May 28;4:15. doi: 10.1186/1753-2000-4-15 (PMC2897776; doi:10.1186/1753-2000-4-15)
Supplement: Additional file 2 — PEP-Screen-ER. Questionnaire used for screening of children at risk for externalizing behavior, teachers' perspective. [file 1753-2000-4-15-S2.PDF]

# Fragebogen für ErzieherInnen von Kindergartenkindern

**IDENTIFIKATIONSNUMMER** (des Kindes):
(Wird durch ErzieherIn vergeben )

Kita Gruppe Kind

| | |
|---|---|
| **GESCHLECHT:** ☐ Junge ☐ Mädchen | **ALTER DES KINDES:** |
| **DIESER FRAGEBOGEN WURDE AUSGEFÜLLT VON :**<br><br>Name | **GEBURTSTAG DES KINDES:** Tag Monat Jahr<br>**HEUTIGES DATUM:** Tag Monat Jahr |

Bitte füllen sie diesen Fragebogen so aus, daß er Ihre Ansichten wiedergibt, auch wenn andere Menschen diese nicht teilen. Zu jeder Frage können Sie gerne auf der Rückseite Bemerkungen oder Kommentare schreiben.

Es folgt eine kurze Liste von Eigenschaften, mit denen man Verhalten von Kindern beschreiben kann. Beantworten Sie bitte für jede Eigenschaft, ob sie jetzt oder innerhalb der letzten 2 Monate bei dem Kind zu beobachten war. Wenn diese Eigenschaft sehr deutlich oder sehr oft zu beobachten war, kreuzen Sie die Ziffer 2 an, wenn die Eigenschaft etwas oder manchmal auftrat, die Ziffer 1, wenn sie für das Kind nicht zutrifft, die Ziffer 0. Beantworten Sie bitte alle Fragen so gut Sie können.

0= nicht zutreffend (soweit bekannt) 1= etwas oder manchmal zutreffend 2= genau oder häufig zutreffend

1. Streitet oder widerspricht viel........ **0 1 2**
2. Wird viel gehänselt........ **0 1 2**
3. Verlangt viel Beachtung........ **0 1 2**
4. Klammert sich an Erwachsene oder ist zu abhängig........ **0 1 2**
5. Kann sich nicht konzentrieren, kann nicht lange aufpassen........ **0 1 2**
6. Kann nicht stillsitzen, ist unruhig oder überaktiv........ **0 1 2**
7. Ist zu furchtsam oder ängstlich........ **0 1 2**
8. Macht Sachen kaputt, die anderen gehören........ **0 1 2**
9. Ist unglücklich, traurig oder niedergeschlagen........ **0 1 2**
10. Ist impulsiv oder handelt ohne zu überlegen........ **0 1 2**
11. Sagt häufig, daß ihm etwas weh tut (ohne bekannte körperliche Ursache)... **0 1 2**
12. Greift andere körperlich an........ **0 1 2**
13. Hat Wutausbrüche oder hitziges Temperament........ **0 1 2**

**Insgesamt gesehen**,
als wie belastend erleben Sie die oben beschriebenen Verhaltensweisen bei diesem Kind?
(Zutreffendes bitte ankreuzen)

☐ gar nicht ☐ ein wenig ☐ ziemlich ☐ sehr

**Glauben Sie,**
daß dieses Kind wegen dieser Probleme eine Beratung oder Behandlung braucht, oder daß Sie selbst deswegen Unterstützung brauchen?

☐ Nein ☐ Ja

**BITTE PRÜFEN SIE, OB SIE ALLE FRAGEN VOLLSTÄNDIG BEANTWORTET HABEN!** **VIELEN DANK !**

**SONSTIGE BEMERKUNGEN ZUM KIND:**

## Angaben bei Nicht-Teilnahme der Eltern an der Befragung

**NATIONALITÄT / SPRACHE:** ☐ Deutsch ☐ Andere

**GRÜNDE FÜR NICHT-TEILNAHME:**

| ANGABEN DER ELTERN | | ANNAHMEN DER ERZIEHERIN |
|---|---|---|
| ☐ | Bedenken wegen Datenschutz | ☐ |
| ☐ | mangelnde Deutschkenntnisse | ☐ |
| ☐ | andere (bitte beschreiben): | ☐ |
